# Indirect comparison of the efficacy and safety of alirocumab and evolocumab on major cardiovascular events: a systematic review and network meta-analysis

**DOI:** 10.3389/fphar.2025.1555508

**Published:** 2025-06-24

**Authors:** Leyu Xu, Ming Lei, Liren Li, Yilei Li, Chunping Gu, Ping Zheng

**Affiliations:** ^1^ Department of Pharmacy, Nanfang Hospital, Southern Medical University, Guangzhou, China; ^2^ Clinical Pharmacy Center, Nanfang Hospital, Southern Medical University, Guangzhou, China

**Keywords:** alirocumab, evolocumab, PCSK9 inhibitors, cardiovascular events, efficacy, safety, network meta-analysis

## Abstract

**Background:**

Alirocumab and evolocumab are proprotein convertase subtilisin/kexin type 9 inhibitors that significantly reduce the relative risk of cardiovascular events. However, the relative efficacy and safety of alirocumab and evolocumab in different patient groups still warrant further indirect comparison. This systematic review and network meta-analysis indirectly compared the efficacy and safety of alirocumab and evolocumab on major cardiovascular events.

**Methods:**

PUBMED, EMBASE, Web of Science, and Cochrane Central Register of Controlled Trials (CENTRAL) databases were comprehensively searched to extract randomized controlled trials (RCTs) regarding alirocumab and evolocumab published from inception to 17 August 2024. The meta-analysis was performed using Software Review Manager 5.4 and R 4.1.0 software.

**Results:**

This network meta-analysis included 26 RCTs with 64,921 patients. Among these, 13 RCTs included patients receiving alirocumab or placebo (n = 13,365) and 13 RCTs included patients receiving evolocumab or placebo (n = 22,048). Compared with the placebo, treatment with alirocumab and evolocumab significantly reduced the relative risk of major adverse cardiovascular and cerebrovascular events (MACCE), myocardial infarction, stroke, and coronary revascularization. Furthermore, alirocumab and evolocumab groups did not show significant differences in MACCE [relative risk (RR): 0.99, 95% confidence interval (CI): 0.88–1.11], cardiovascular death (RR: 0.83, 95% CI: 0.65–1.06), myocardial infarction (RR: 0.87, 95% CI: 0.74–1.03), stroke (RR: 0.96, 95% CI: 0.71–1.29), coronary revascularization (RR: 0.88, 95% CI: 0.77–1.01), and any adverse event (RR: 0.91, 95% CI: 0.76–1.09). Moreover, the all-cause mortality rates were lower for patients treated with alirocumab compared to those treated with evolocumab (RR: 0.84, 95% CI: 0.70–1.00), but the difference was not statistically significant.

**Conclusion:**

Alirocumab and evolocumab demonstrated comparable efficacy in reducing the relative risk of major cardiovascular events. The all-cause mortality rates were lower in patients treated with alirocumab compared to those treated with evolocumab but the differences were not statistically significant, probably due to heterogeneity in the sample size and follow-up duration between different studies. Both drugs exhibited comparable safety profiles.

**Systematic Review Registration:**

https://www.crd.york.ac.uk/PROSPERO/myprospero, identifier CRD42024505327.

## 1 Introduction

Atherosclerotic cardiovascular disease (ASCVD) is the leading cause of death globally and low-density lipoprotein cholesterol (LDL-C) is a key, modifiable risk factor of ASCVD events (Diao et al., 2024). Reducing LDL-C levels is the primary target of lipid-lowering treatment to prevent and manage ASCVD (Mhaimeed et al., 2024). Statins are considered as the first-line therapeutics for reducing elevated LDL-C levels according to the current clinical practice guidelines. Proprotein convertase subtilisin/kexin type 9 inhibitors (PCSK9i) are considered as second line therapy for patients that do not achieve optimal goals of statin therapy (Grundy et al., 2019; Bhatia et al., 2024). Statins are effective in reducing LDL-C levels in only approximately 50% of patients with dyslipidemia (Ray et al., 2021; van de Borne et al., 2024). More than 50% of patients with familial hypercholesterolemia (FH) fail to achieve the LDL-C treatment goals even after receiving the highest tolerated statin dose (Schreuder et al., 2023). Furthermore, there is a strong consensus that statin intolerance leads to poor adherence and persistence with statins, and contributes to worsening cardiovascular outcomes (Banach et al., 2023). Therefore, patients with statin tolerance require alternate LDL-C targeting therapies.

PCSK9i are effective in lowering LDL-C levels and reducing the risk of ASCVD events by competitively inhibiting the binding of PCSK9 to the LDL receptors (LDLRs), thereby maintaining higher hepatic LDLR density and enhancing LDL-C clearance (Hummelgaard et al., 2023). Several humanized monoclonal antibodies that selectively target PCSK9 receptors have been developed for clinical practice. Alirocumab and evolocumab are the two most extensively studied PCSK9i that have shown high safety and efficacy in managing patients with hypercholesterolemia and ASCVD. These two antibodies bind to PCSK9 at sites overlapping with the binding site of LDLR, thereby effectively outcompeting the interaction between PCSK9 and LDLR. PCSK9i such as inclisiran and toralizumab are only approved for treating primary hypercholesterolemia and mixed dyslipidemia, but not for reducing cardiovascular risk. According to the 2021 European Society of Cardiology guidelines, early combination therapy with a PCSK9i is recommended for patients who fail to achieve their lipid goals with a maximum tolerated dose of a statin and ezetimibe (Visseren et al., 2022). However, further evidence is required regarding the long-term safety and efficacy of evolocumab and alirocumab in reducing ASCVD events because of the high yearly cost for these two drugs at £45,279 and £46,375, respectively (Michaeli et al., 2022).

Large randomized controlled trials (RCTs) have confirmed that PCSK9i are highly effective in lowering LDL-C levels and are associated with significant beneficial outcomes, including a reduction in all-cause mortality, cardiovascular events, and cardiovascular mortality (Sabatine et al., 2017; Schwartz et al., 2018). However, two large RCTs evaluating the use of alirocumab (ODYSSEY OUTCOMES trial) and evolocumab (FOURIER trial) enrolled patients with distinct clinical profiles, resulting in contradictory conclusions. According to currently available evidence, alirocumab demonstrates better outcomes in subjects with a higher risk of ASCVD, whereas evolocumab shows higher efficacy in patients with heterozygous familial hypercholesterolemia. However, direct comparison between these two agents has not been performed in clinical trials. An indirect comparison of the safety and efficacy of alirocumab and evolocumab based on a systematic review and network meta-analyses of RCTs was performed in 2021 and demonstrated comparable safety and efficacy profiles despite heterogeneity in the study populations (Guedeney et al., 2021). In recent years, several new large RCTs of alirocumab or evolocumab have been reported but have not been evaluated through meta-analyses. While recent studies have demonstrated the robust efficacy of alirocumab and evolocumab in reducing major cardiovascular events across various patient populations, the relative efficacy and safety of these two agents still warrant further indirect comparison. Therefore, in this network meta-analysis, we compared the efficacy and safety of alirocumab and evolocumab on major cardiovascular events by indirectly evaluating the results of RCTs, including those from newer RCTs. We included RCTs with data on outcomes such as major adverse cardiovascular and cerebrovascular events (MACCE), all-cause mortality, cardiovascular deaths, myocardial infarction, stroke, and coronary revascularization.

## 2 Methods

### 2.1 Protocol registration

The protocol for this systematic review was registered in the PROSPERO database (No. CRD42024505327). This study was approved by the Ethics Committee of Nanfang Hospital (Approval No. NFEC-2023-208).

We searched the PUBMED, EMBASE, Web of Science, and Cochrane Central Register of Controlled Trials databases from inception to 17 August 2024, using the following search terms: ‘alirocumab’ OR ‘evolocumab’ AND ‘randomized controlled trial’. The searches were not limited by any publication or language restrictions.

### 2.2 Selection criteria and outcomes

We included only RCTs that compared alirocumab or evolocumab with placebo in patients with dyslipidemias or cardiovascular disease and reported cardiovascular events and other adverse events. To reduce small-study effects and increase the reliability of our findings, we specifically included studies with a minimum of 100 participants and a follow-up period of at least 8 weeks.

This study focused only on alirocumab and evolocumab. RCTs comparing alirocumab or evolocumab with other lipid-lowering medications but lacking a placebo group were also excluded from the analysis.

MACCE was the primary composite efficacy outcome and defined as the occurrence of all-cause mortality, cardiovascular death, myocardial infarction, stroke, or coronary revascularization. Individual components of MACCE, namely, all-cause mortality, cardiovascular death, myocardial infarction, stroke, and coronary revascularization were included as secondary efficacy outcomes. Safety endpoints encompassed any reported adverse events.

### 2.3 Data extraction and analysis

Two researchers (CG and LX) independently extracted data from eligible studies using a pre-specified data collection form. The extracted data included study characteristics (authors, year of publication, and number of patients), study design (double-blind or open-label), characteristics of the patients enrolled, treatment protocols (name, dosage, and follow-up time), as well as the efficacy and safety outcomes.

One of the authors, LX, independently assessed the risk of bias according to the criteria outlines in the Cochrane Handbook for systematic reviews of interventions for RCTs. The methodological quality of all studies included in the analysis was assessed using the Review Manager (RevMan, Version 5.4; The Cochrane Collaboration). This assessment included random sequence generation, allocation concealment, blinding of participants and personnel, blinding of outcome assessment, incomplete outcome data, and selective reporting. The risk of bias was judged as low, unclear, and high.

Publication bias was evaluated with a combination of methods using R, including visual analysis with funnel plots and statistical tests like Begg’s test, Egger’s test, and Thompson-Sharp’s test. The threshold for statistical significance was set at p < 0.05.

A league table was generated for all pairwise comparisons in the meta-analysis using the odds ratios (OR) and 95% confidence intervals (95% CI) to directly compare the direction and magnitude of treatment effects. The analysis was performed using Software Review Manager 5.4 (RRID:SCR_003581) and R 4.1.0 software (RRID:SCR_001905).

## 3 Results

### 3.1 Literature search results

**FIGURE 1:**
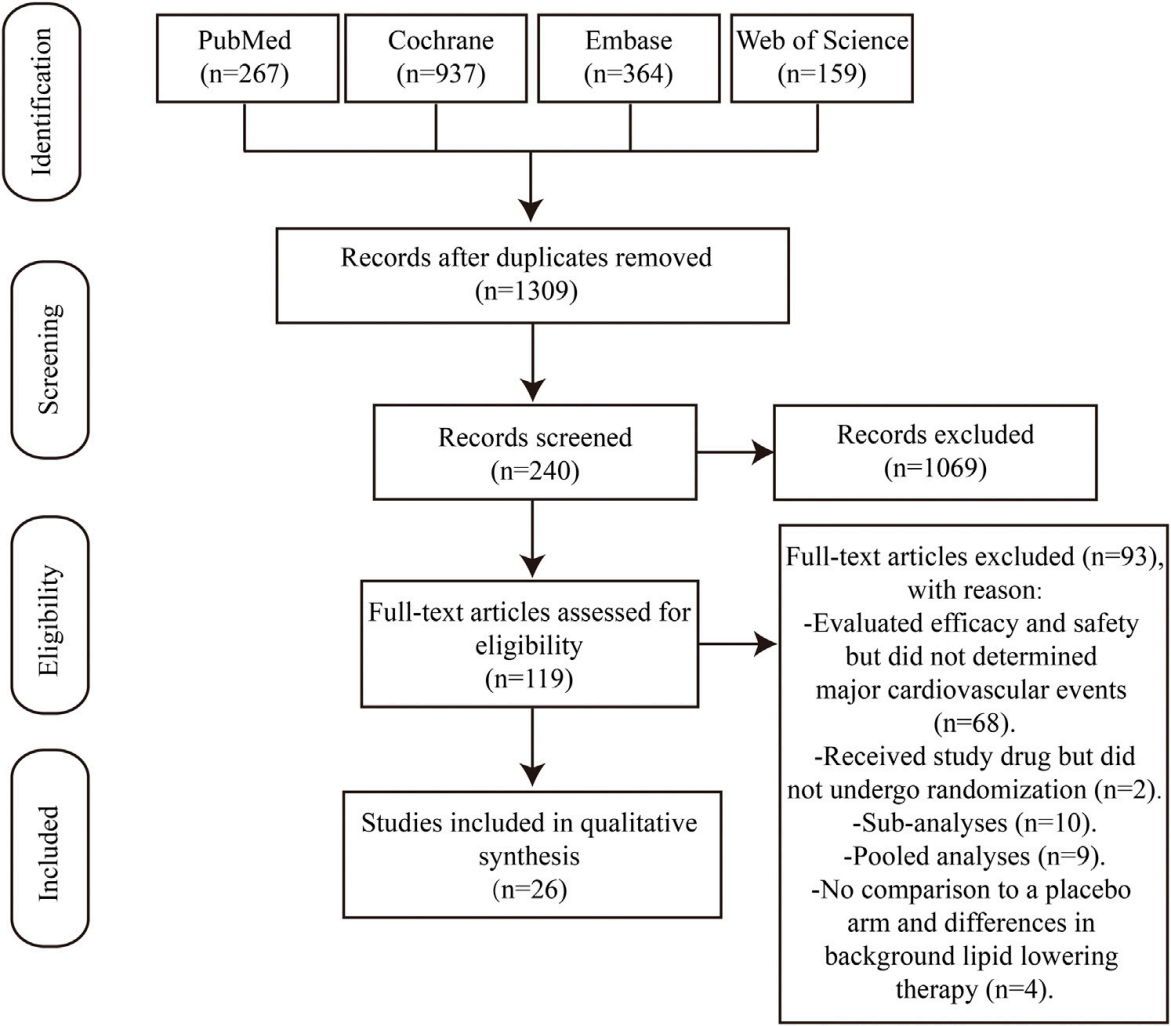
PRISMA flow diagram. PRISMA, Preferred Reporting Items for Systematic Reviews and Meta-analysis. A total of 1727 articles were retrieved, and 26 studies were selected for network meta-analysis.

**FIGURE 2:**
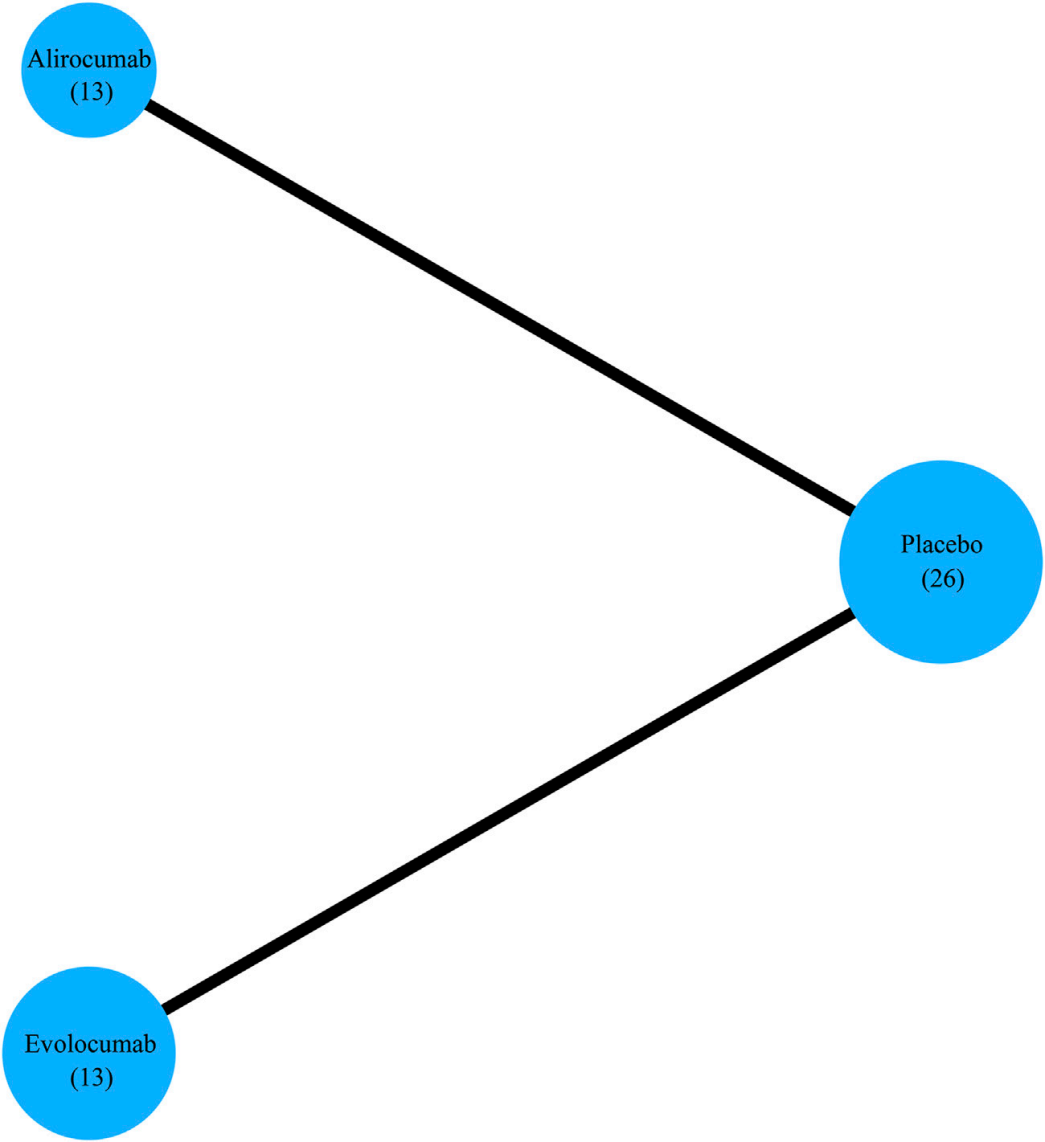
Network diagram shows the total number of RCTs analyzed for each treatment arm to evaluate efficacy endpoints.

Based on searches in the four electronic databases, we initially included 1,727 studies for this updated systematic review. After removing duplicate publications, 1,309 articles were selected for further evaluation. Among these, we eliminated 1,069 articles that were reviews, comments, short reports, and case studies. Furthermore, 92 articles were excluded because of the following reasons: (i) efficacy and safety of the study drug was evaluated but major cardiovascular events were not analyzed (n = 68); (ii) patients received the study drug but did not undergo randomization (n = 2); (iii) sub-analyses (n = 10); (iv) pooled analyses (n = 9); (v) absence of comparison with a placebo arm and differences in the background lipid-lowering therapy (n = 4). Finally, this study enrolled 26 studies that met the inclusion criteria (Figure 1), including 13 RCTs comparing alirocumab with placebo and 13 RCTs comparing evolocumab with placebo. Overall, the data included 64,921 patients, with 24,851 patients allocated to alirocumab and 40,070 patients allocated to evolocumab. The corresponding network diagram is shown in Figure 2.

### 3.2 Overview of study and patient characteristics

This study enrolled data from 26 studies with 64,921 participants (107–27,564 patients per study) (Kastelein et al., 2015; Robinson et al., 2015; Kereiakes et al., 2015; Ginsberg et al., 2016; Roth et al., 2016; Stroes et al., 2016; Teramoto et al., 2016; Leiter et al., 2017; Schwartz et al., 2018; Koh et al., 2018; Teramoto et al., 2019; Räber et al., 2022; Koren et al., 2012; Giugliano et al., 2012; Blom et al., 2014; Hirayama et al., 2014; Robinson et al., 2014; Koren et al., 2014; Sabatine et al., 2015; Raal et al., 2015; Nicholls et al., 2016; Kiyosue et al., 2016; Sabatine et al., 2017; Koren et al., 2019; Koskinas et al., 2019). The median age of the participants was similar across all studies (Table 1). The doses of alirocumab and evolocumab varied. The dosage for alirocumab were 75 mg Q2W, 150 mg Q2W, 150 mg Q4W, or 300 mg Q4W, and the dosage for evolocumab were 70 mg Q2W, 105 mg Q2W, 140 mg Q2W, 280 mg Q4W, 350 mg Q4W, or 420 mg Q4W (Table 1). Figure 2 shows a network of eligible treatment comparisons for major cardiovascular events.

**TABLE 1:** Study and patient characteristics of included studies.

Study	Author and year	Subjects	Study design	No.	Follow-up period in weeks	Median age, y	Male, %	White, %	Intervention	Diabetes mellitus, %	On statin, %	Key outcomes
ODYSSEY FH I	Kastelein et al. (2015)	patients with HeFH and inadequate LDL-C control on maximally tolerated LLT	a randomized, double-blind, placebo-controlled trial	486	78 weeks (total)	ALI: 52.1; Placebo: 51.7	56.4	91.4	ALI: 75 mg Q2W	11.7	100	①②③④⑤⑥⑦
ODYSSEY FH II	Kastelein et al. (2015)	patients with HeFH and inadequate LDL-C control on maximally tolerated LLT	a randomized, double-blind, placebo-controlled trial	249	78 weeks (total)	ALI: 53.2; Placebo: 53.2	52.6	98	ALI: 75 mg Q2W	4	100	①②③④⑤⑥⑦
ODYSSEY LONG TERM	Robinson et al. (2015)	patients with HeFH or with established coronary heart disease or a coronary heart disease risk equivalent	a phase 3, randomized, double-blind, placebo-controlled, parallel-group, multinational study	2,338	78 weeks (total)	ALI: 60.4; Placebo: 60.6	62	92.7	ALI: 150 mg Q2W	34.6	99.9	①②③④⑤⑥⑦
ODYSSEY COMBO I	Kereiakes et al. (2015)	high cardiovascular risk patients on maximally tolerated statin therapy	a randomized, double-blind, placebo-controlled trial	316	52 weeks (total)	ALI: 63.0; Placebo: 63.0	65.8	81.6	ALI: 75 mg Q2W	43	99.7	①②③④⑤⑥⑦
ODYSSEY HIGH FH	Ginsberg et al. (2016)	patients with HeFH and LDL-C levels of 160 mg/dL or Higher	a randomized, double-blind, placebo-controlled trial	107	78 weeks (total)	ALI: 49.8; Placebo: 52.1	53.3	87.9	ALI: 150 mg Q2W	15	100	①②③④⑤⑥⑦
ODYSSEY CHOICE I	Roth et al. (2016)	patients with hypercholesterolemia at moderate-to-very-high cardiovascular risk	a randomized, double-blind, placebo-controlled trial	803	56 weeks (total)	Not statin (ALI 75 mg Q2W: 59.3, 300 mg Q4W: 59.2, Placebo: 59.4); Statin (ALI 75 mg Q2W: 60.7, 300 mg Q4W: 61.6, Placebo: 61.6)	57.5	87.3	ALI: 75 mg Q2W or 300 mg Q4W	27	68.1	①②③④⑦
ODYSSEY CHOICE II	Stroes et al. (2016)	patients with hypercholesterolemia receiving fenofibrate or ezetimibe or diet alone	a randomized, double-blind, placebo-controlled trial	233	32 weeks (total)	ALI (75 mg Q2W: 62.5; 150 mg Q4W: 64.2); Placebo: 63.1	55.8	94	ALI: 150 mg Q4W or 75 mg Q2W	16.3	0	①②③④⑤⑥⑦
ODYSSEY JAPAN	Teramoto et al. (2016)	patients with HeFH or at high cardiovascular risk with hypercholesterolemia not adequately controlled with statins	a randomized, double-blind, placebo-controlled trial	216	60 weeks (total)	ALI: 60.3; Placebo: 61.8	60.6	NA	ALI: 75 mg Q2W with increase to 150 mg if week 8 LDL-C≥2.6/3.1 mmol/L	68.5	100	①②③④⑥⑦
ODYSSEY DM-INSULIN	Leiter et al. (2017)	insulin-treated individuals with type 1 or type 2 diabetes and high cardiovascular risk	a phase IIIb, randomized, double-blind placebo-controlled, parallel-group, multicenter trial	517	24 weeks (total)	T2D (ALI: 63.9; Placebo: 64.0); T1D (ALI: 54.9; Placebo: 58.5)	55.1	90.5	ALI: 75 mg Q2W, with blinded dose increase to 150 mg every 2 weeks at week 12 if week 8 LDL-C≥1.8 mmol/L	100	74.9	②③④⑦
ODYSSEY OUTCOMES	Schwartz et al. (2018)	patients with acute coronary syndrome receiving high-intensity statin therapy	A randomized, double-blind, placebo-controlled trial	18,924	208 weeks (total)	ALI: 58.5; Placebo: 58.6	75	79.4	ALI: 75 mg or 150 mg Q2W	29.1	100	①②③④⑤⑥⑦
ODYSSEY KT	Koh et al. (2018)	patients with hypercholesterolemia, at high cardiovascular risk, and on maximally tolerated statin	a randomized, double-blind, placebo-controlled trial	199	32 weeks (total)	ALI: 60.1; Placebo: 61.2	82.4	NA	ALI: 75 mg or 150 mg Q2W	35.2	100	①②③④⑤⑥⑦
ODYSSEY NIPPON	Teramoto et al. (2019)	hypercholesterolemic patients on non-statin lipid-lowering therapy or lowest strength dose of statin	a randomized, double-blind, placebo-controlled trial	163	12 weeks (total)	ALI (150 mg Q4W: 62.6; 150 mg Q2W: 63.6); Placebo: 64.6	63.2	NA	ALI: 150 mg Q4W or 150 mg Q2W	55.2	34.4	①②③④⑥⑦
PACMAN-AMI	Räber et al. (2022)	patients with acute myocardial infarction	a randomized, double-blind, placebo-controlled trial	300	52 weeks (total)	ALI: 58.4; Placebo: 58.6	81.3	NA	ALI: 150 mg Q2W	10.3	12.3	①②③④⑤⑥⑦
MENDEL	Koren et al. (2012)	patients with serum LDL-C concentrations of 2.6 mmol/L or greater but less than 4.9 mmol/L	a randomized, double-blind, placebo and ezetimibe-controlled	361	12 weeks (total)	Q2W (EVO 70 mg: 50.9; 105 mg: 48.3; 140 mg: 52.8; Placebo: 52.5); Q4W (EVO 280 mg: 49.3; 350 mg: 50.9; 420 mg: 50.1; Placebo: 50.7); Ezetimibe 10 mg QD: 50.0	34.2	78.6	EVO: Q2W: 70 mg or 105 mg 140 mg; Q4W: 280 mg or 350 mg or 420 mg	0.2	0	①②③④⑤⑥⑦
LAPLACE-TIMI 57	Giugliano et al. (2012)	patients with LDL-C greater than 2.2 mmol/L on a stable dose of statin (with or without ezetimibe)	a randomized, double-blind, placebo-controlled trial	631	12 weeks (total)	Q2W (EVO 70 mg: 62.0; 105 mg: 59.0; 140 mg: 63.5; Placebo: 61.0); Q4W (EVO 280 mg: 61.0; 350 mg: 64.0; 420 mg: 63.0; Placebo: 63.0)	49.1	89	EVO: Q2W: 70 mg or 105 mg 140 mg; Q4W: 280 mg or 350 mg or 420 mg	16.3	99.4	①②③④⑤⑥⑦
DESCARTES	Blom et al. (2014)	patients with hyperlipidemia	a phase 3, randomized, double-blind, placebo-controlled trial	901	52 weeks (total)	EVO: 55.9; Placebo: 56.7	47.7	80.4	EVO: 420 mg Q4W	11.5	87.7	①②③④⑦
YUKAWA	Hirayama et al. (2014)	hypercholesterolemic, statin-treated Japanese patients at high cardiovascular risk	a randomized, double-blind, placebo-controlled trial	307	12 weeks (total)	Q2W (EVO 70 mg: 64.1; 140 mg: 60.8; Placebo: 60.2); Q4W (EVO 280 mg: 61.6; 420 mg: 61.3; Placebo: 60.9)	62.9	NA	EVO: Q2W: 70 mg or 140 mg; Q4W: 280 mg or 420 mg	38.1	100	①②③⑥⑦
LAPLACE-2	Robinson et al. (2014)	screening LDL-C levels of 150 mg/dL or greater (no statin at screening), 100 mg/dL or greater (non-intensive statin at screening), or 80 mg/dL or greater (intensive statin at screening) and fasting triglyceride levels of 400 mg/dL or less	a randomized, double-blind, placebo-and ezetimibe-controlled trial	1,675	12 weeks (total)	EVO: 59.6; Placebo: 59.9; Ezetimibe: 60.8	54.2	94	EVO: 420 mg Q4W or 140 mg Q2W	15.5	0	①②⑦
MENDEL-2	Koren et al. (2014)	patients with fasting LDL-C≥100 and≤190 mg/dL and Framingham risk scores≤10%	a randomized, double-blind, placebo-and ezetimibe-controlled trial	460	12 weeks (total)	Q2W (EVO 140 mg: 53; Placebo: 54; Ezetimibe: 54); Q4W (EVO 420 mg: 53; Placebo: 53; Ezetimibe: 53)	31.1	83.1	EVO: 420 mg Q4W or 140 mg Q2W	0.16	0	①②③⑦
OSLER	Sabatine et al. (2015)	patients with hyperlipidemia and mixed dyslipidemia	An open-label, randomized, controlled study	4,465	44.4 weeks (median)	EVO: 57.8; Standard-Therapy: 58.2	50.5	85.7	EVO: 140 mg Q2W or 420 mg Q4W	13.4	86.5	①②③④⑤⑥⑦
RUTHERFORD-2	Raal et al. (2015)	patients with HeFH	a randomized, double-blind, placebo-controlled trial	329	12 weeks (total)	Q2W (EVO 140 mg: 52.6; Placebo: 51.1); Q4W (EVO 420 mg: 51.9; Placebo: 46.8)	57.8	89	EVO:420 mg Q4W or 140 mg Q2W	0	100	①②⑦
GLAGOV	Nicholls et al. (2016)	patients with angiographic coronary disease treated with statins	a randomized, double-blind, placebo-controlled trial	968	78 weeks (total)	EVO: 59.8; Placebo: 59.8	72.2	93.8	EVO: 420 mg Q4W	20.9	98.6	①②③④⑤⑥⑦
YUKAWA II	Kiyosue et al. (2016)	patients with hyperlipidemia or mixed dyslipidemia and high cardiovascular risk	a randomized, double-blind, placebo-controlled trial	404	12 weeks (total)	EVO: 62; Placebo: 61	60.4	NA	EVO: 420 mg Q4W or 140 mg Q2W	48.8	100	①⑤⑦
FOURIER	Marc S. Sabatine, 2017	patients with atherosclerotic cardiovascular disease and LDL-C≥70 mg/dL	a randomized, double-blind, placebo-controlled trial	27,564	114.4 weeks (median)	EVO: 62.5; Placebo: 62.5	75	85.1	EVO: 420 mg Q4W or 140 mg Q2W	36.5	100	①②③④⑤⑥⑦
OSLER-1	Koren et al. (2019)	patients with hypercholesterolemia	a randomized, open-label, controlled study	1,697	52 weeks (total)	EVO: 57.1; SOC: 57.6	46.8	73.2	EVO: 420 mg Q4W	14.1	68.1	①⑦
EVOPACS	Koskinas et al. (2019)	patients with acute coronary syndromes	an investigator-initiated, prospective, randomized, double-blind, placebo-controlled, parallel-group, phase III trial	308	8 weeks (total)	EVO: 60.5; Placebo: 61.0	81.5	NA	EVO: 420 mg Q4W	15.3	21.8	①②③④⑤⑥⑦

HeFH, heterozygous familial hypercholesterolaemia; ALI, alirocumab; EVO, evolocumab; Q2W, once every 2 weeks; Q4W, once every 4 weeks; QD, once per day; LLT, lipid-lowering therapy; T1D, Type 1 Diabetes; T2D, Type 2 Diabetes; SOC, standard of care; NA, not available; ①, cardiovascular events; ②, all-cause mortality; ③, cardiovascular death; ④, myocardial infarction; ⑤, stroke; ⑥, coronary revascularization; ⑦, any adverse events.

### 3.3 Risk of bias for the included studies

The quality of included studies was assessed using the Cochrane Risk of Bias Tool. A low risk of bias was identified across several domains because all the included studies were randomized and majority of the studies were double-blinded for both participants and personnel. There were fewer instances of selective reporting. However, approximately half of the studies did not adequately address random sequence generation and allocation concealment, which are indicators of selection bias.

**FIGURE 3:**
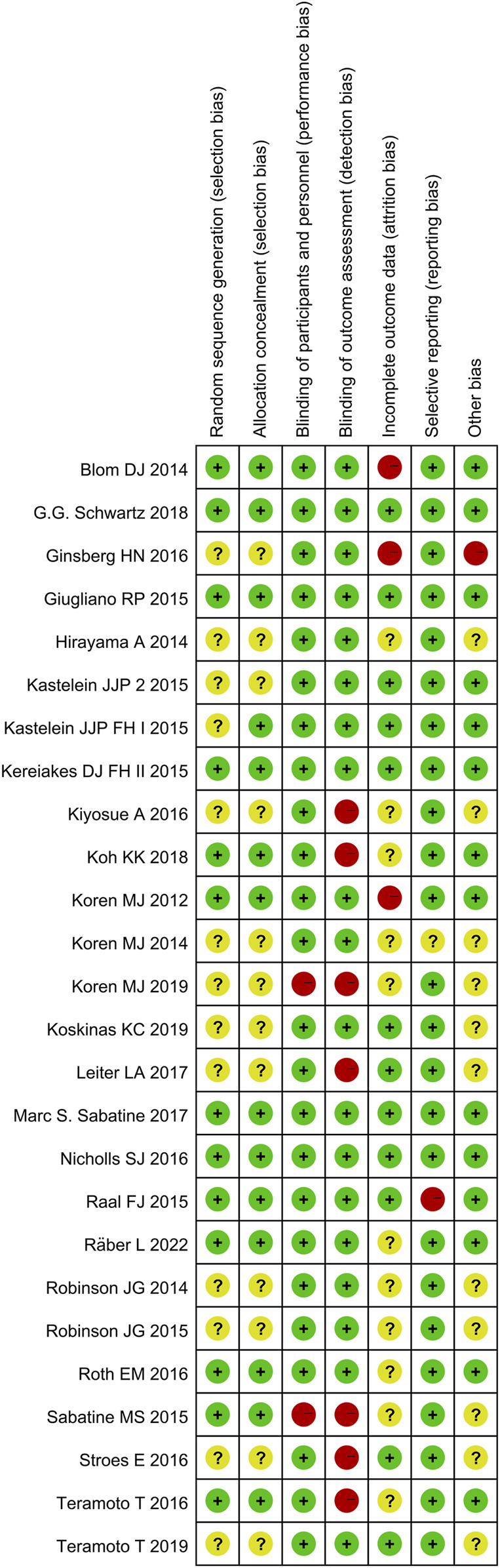
Risk of bias summary.

**FIGURE 4:**
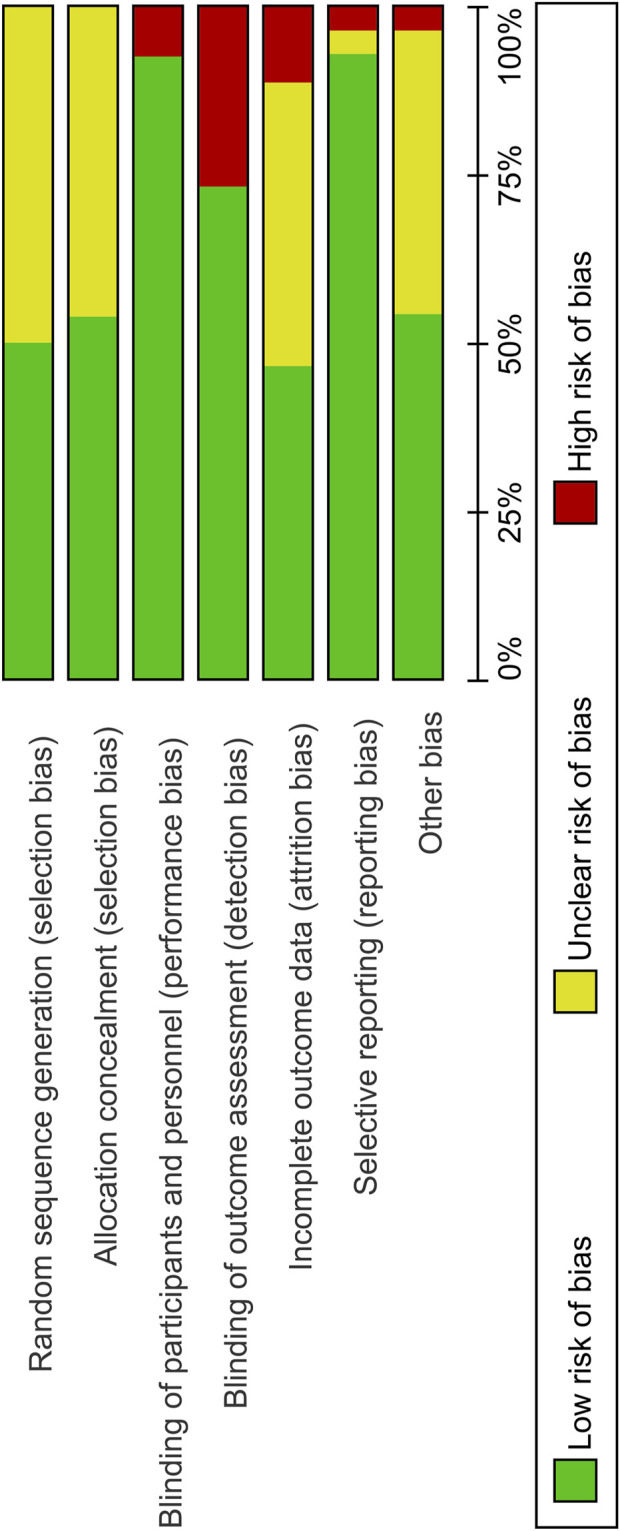
Risk of bias graph.

The risk of bias summary and graph for the included studies are shown in Figures 3, 4, respectively.

### 3.4 Efficacy endpoints

#### 3.4.1 Major adverse cardiac and cerebrovascular events

**FIGURE 5:**
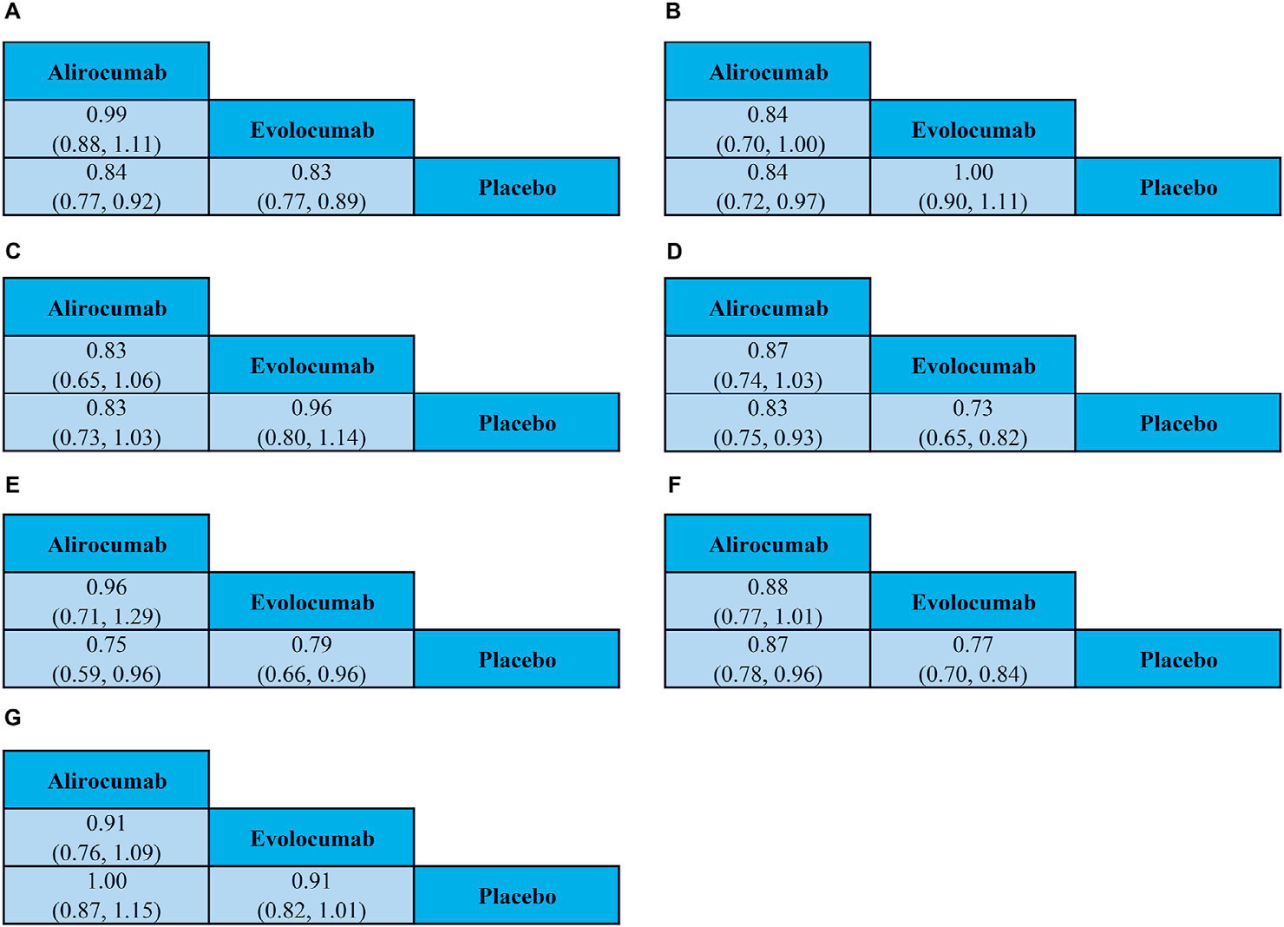
League table highlights the main findings of the outcome analysis. **(A)** Major adverse cardiac and cerebrovascular events; **(B)** All-cause mortality; **(C)** Cardiovascular death; **(D)** Myocardial infarction; **(E)** Stroke; **(F)** Coronary revascularization; **(G)** Any adverse events. For each comparison, odds ratios and 95% confidence intervals are provided.

This meta-analysis included 25 trials that reported MACCE. As shown in Figure 5A, the risk of MACCE was significantly reduced in patients treated with alirocumab (RR: 0.84, 95% CI: 0.77–0.92) or evolocumab (RR: 0.83, 95% CI: 0.77–0.89). Furthermore, there were no significant differences in the risk of MACCE between patients receiving the two drugs (RR: 0.99, 95%CI: 0.88–1.11).

#### 3.4.2 All-cause mortality

Twenty-four of the included evaluated all-cause mortality outcomes. As shown in Figure 5B, patients treated with alirocumab demonstrated reduced risk of all-cause mortality (RR: 0.84, 95% CI: 0.72–0.97), but those treated with evolocumab did not show statistically significant decrease in the all-cause mortality events (RR: 1.00, 95% CI: 0.90–1.11). Furthermore, comparative analysis demonstrated that the risk of all-cause mortality was lower in those treated with alirocumab compared to those treated with evolocumab (RR: 0.84, 95% CI: 0.70–1.00). However, since the 95% confidence interval included the null value (1.00), the observed differences did not demonstrate statistical significance and cannot be considered as conclusive evidence of superiority.

#### 3.4.3 Cardiovascular death

Cardiovascular death outcomes were reported in 24 studies. As shown in Figure 5C, we did not observe significant reduction in the cardiovascular death events among patients receiving alirocumab (RR: 0.83, 95% CI: 0.73–1.03) or evolocumab (RR: 0.96, 95% CI: 0.80–1.14). Furthermore, there were no significant differences in the risk of cardiovascular death between patients receiving alirocumab or evolocumab (RR: 0.83, 95% CI: 0.65–1.06).

#### 3.4.4 Myocardial infarction

Myocardial infarction outcomes were reported in 19 studies. As shown in Figure 5D, the risk of myocardial infarction was reduced in patients treated with alirocumab (RR: 0.83, 95% CI: 0.75–0.93) or evolocumab (RR: 0.73, 95% CI: 0.65–0.82). However, we did not observe any statistically significant differences in the risk of myocardial infarction risk between patients receiving the two drugs (RR: 0.87, 95% CI: 0.74–1.03).

#### 3.4.5 Stroke

Stroke outcomes were reported in 15 studies. As shown in Figure 5E, the risk of stroke was lower in those treated with alirocumab (RR: 0.75, 95% CI: 0.59–0.96) or evolocumab (RR: 0.79, 95% CI: 0.66–0.96). However, we did not observe any statistically significant difference in the risk of stroke between patients receiving the two drugs (RR: 0.96, 95% CI: 0.71–1.29).

#### 3.4.6 Coronary revascularization

Coronary revascularization outcomes were evaluated by 18 studies. As shown in Figure 5F, patients treated with alirocumab (RR: 0.87, 95%: CI 0.78–0.96) or evolocumab (RR: 0.77, 95% CI: 0.70–0.84) were associated with a lower risk of coronary revascularization. However, the risk of coronary revascularization was comparable between those treated with alirocumab or evolocumab (RR 0.88, 95% CI 0.77–1.01).

### 3.5 Safety endpoints

Safety outcomes were reported in all the included studies. As shown in Figure 5G, there was no significant difference in the incidence rates of adverse events between those treated with evolocumab or alirocumab. Furthermore, there were no significant differences in the risk of adverse events leading to treatment discontinuation between patients treated with evolocumab or alirocumab (RR: 0.91, 95% CI: 0.76–1.09).

### 3.6 Analysis of publication bias

**FIGURE 6:**
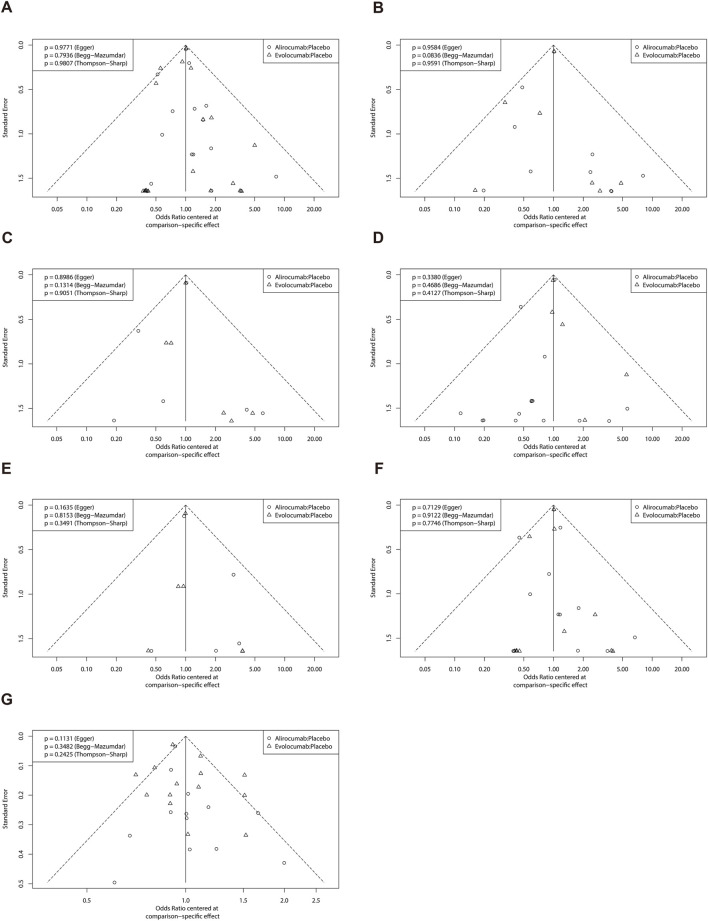
Funnel plot of efficacy and safety endpoints. **(A)** Major adverse cardiac and cerebrovascular events; **(B)** All-cause mortality; **(C)** Cardiovascular death; **(D)** Myocardial infarction; **(E)** Stroke; **(F)** Coronary revascularization; **(G)** Any adverse events.

The funnel plots for all the studies are shown in Figures 6A–G. These plots were visually symmetrical. The results of Begg’s test, Egger’s test, and Thompson-Sharp’s test did not demonstrate any evidence of publication bias (P ≥ 0.05).

## 4 Discussion

In this systematic review and meta-analysis, we analyzed data from 26 separate RCTs to compare the efficacy and safety of alirocumab and evolocumab in reducing major cardiovascular events among ASCVD patients. Both alirocumab and evolocumab showed comparable efficacy in reducing MACCE, cardiovascular death, myocardial infarction, stroke, and coronary revascularization. The all-cause death rates were lower in patients treated with alirocumab compared to those treated with evolucumab, but the differences were not statistically significant. Furthermore, there were not significant differences between the two drugs in terms of safety endpoints. To the best of our knowledge, this is the first network meta-analysis that indirectly evaluated the effectiveness and safety of alirocumab and evolocumab on major cardiovascular events.

PCSK9i reduce the risk of developing ASCVD through LDLR-dependent and LDLR-independent mechanisms, including inflammation, plaque formation, and thrombosis (Luquero et al., 2021; Hummelgaard et al., 2023). In 2015, two antibody-based PCSK9i─alirocumab and evolocumab─were approved by the FDA and the European Medicines Agency (EMA) (González-Lleó et al., 2024) to reduce cholesterol levels. Several large RCTs have investigated the efficacy and safety of alirocumab (Schwartz et al., 2018; Bittner et al., 2020) and evolocumab (Sabatine et al., 2015; Sabatine et al., 2017) in reducing cardiovascular events. Furthermore, one study performed indirect comparative analysis of the efficacy and safety of alirocumab versus evolocumab (Guedeney et al., 2021), but their effects on major cardiovascular events are not clear. Therefore, we extracted data from large trials in which cardiovascular events and other adverse events were reported, and aimed to improve the evaluation and reporting of the efficacy and safety profiles of alirocumab and evolocumab on major cardiovascular events.

Systematic meta-analysis of data from 26 RCTs demonstrated that alirocumab or evolocumab were associated with a significant reduction of MACCE, myocardial infarction, stroke, or coronary revascularization compared to the placebo control. The FOURIER trial showed that evolocumab significantly improved the composite cardiovascular outcomes among participants with a baseline LDL-C level of ≥70 mg/dL and those with an average baseline LDL-C level of 90 mg/dL (Blom DJ, et al., 2014). The ODYSSEY OUTCOMES trial showed that alirocumab significantly reduced the risk of cardiovascular outcomes in participants with a baseline LDL cholesterol level of ≥100 mg/dL (Schwartz et al., 2018). The current trial showed that both alirocumab and evolocumab were highly effective in reducing major cardiovascular events in the high-risk ASCVD patients. Previous studies reported continued cardiovascular benefit even when LDL cholesterol levels were reduced to levels below the current target range of 20–25 mg/dL (Writing Committee et al., 2016; Landmesser et al., 2017; Sabatine, 2017).

Lowering blood cholesterol levels, especially LDL-C, can significantly reduce the risk of ASCVD, including coronary artery disease, one of the leading causes of death worldwide (Hummelgaard et al., 2023). PCSK9 binds to the epidermal growth factor-like repeat A domain of the LDLR. This interaction is increased by about150-fold under acidic pH conditions in the endosomes. This increased affinity directs the LDLR-PCSK9 complex to lysosomal degradation and prevents its recycling to the cell surface. Heparan sulfate proteoglycans act as co-receptors of PCSK9 on the surface of the hepatocytes and promote depletion of LDLR, thereby elevating plasma LDL-C levels (Park et al., 2024). PCSK9 also contributes to the development of cardiovascular disease in a LDLR-dependent or LDLR-independent manner, and is involved in the promotion of inflammation, plaque development, and thrombosis (Luquero et al., 2021; D’Onofrio et al., 2023). In macrophages, PCSK9 upregulates scavenger receptors (SRA, CD36, and LOX-1), and promotes uptake of oxidized LDL and secretion of pro-inflammatory cytokines. PCSK9i mitigate thrombosis risk by reducing platelet activation and neutrophil extracellular trap formation (Hummelgaard et al., 2023). Large cohort RCTs have demonstrated the effectiveness of these two drugs in lowering the risk of composite cardiovascular outcomes, including cardiovascular death, myocardial infarction, stroke, and unstable angina (Sabatine et al., 2017; Schwartz et al., 2018). The efficacy and safety of alirocumab and evolocumab has been further validated in real-world settings. A multicenter observational study involving 798 patients confirmed that both drugs were safe and effective in clinical practice and demonstrated high treatment adherence and persistence, with most patients achieving the guideline-recommended LDL-C target levels (Gargiulo et al., 2023). Furthermore, intensive and early lipid-lowering therapy with PCSK9i was safe and effective in acute coronary syndrome (ACS) patients (strike early-strike strong strategy) and associated with reduced residual cardiovascular risk (Gargiulo et al., 2024). Previous meta-analyses have primarily assessed the efficacy of PCSK9i through direct comparisons with the placebo (Dicembrini et al., 2019; Imbalzano et al., 2023). Few meta-analyses have indirectly compared alirocumab with evolocumab (Guedeney et al., 2021; Wang et al., 2022). This meta-analysis aimed to update the comparison of the efficacy and safety profiles of alirocumab and evolocumab by incorporating data from several new RCTs that have been published regarding the efficacy and safety of these two PCSK9i.

Regarding all-cause mortality, our findings were consistent with those reported in an earlier meta-analysis, which indirectly compared the efficacy and safety of these two agents (Guedeney et al., 2021). Alirocumab was associated with a lower relative risk of all-cause mortality compared to evolocumab but the differences were not statistically significant. This may be attributed to differences in the sample size and follow-up duration between different studies. According to the cochrane handbook for systematic reviews of interventions, we enrolled a minimum of 100 participants in order to decrease the standard error. However, the sample size with a minimum of 100 participants can influence study outcomes by introducing biases. In addition, the duration of the study is one of the important study design description of each included study. We collected the varying treatment durations (ranging from 8 to 208 weeks) to facilitate assessment of the risk of bias in each included study. Furthermore, this probably due to heterogeneity in the design of the ODYSSEY OUTCOMES trial. In the ODYSSEY OUTCOMES trial, while key secondary endpoints were examined via a hierarchical statistical approach to control type I error. The analysis of all-cause mortality was outside of this formal hierarchical testing approach. Therefore, the results were considered exploratory. Our data showed that all-cause mortality rates between the two drugs were not statistically significant. This may be a result of including clinical trials that assessed both cardiovascular events and adverse events. Furthermore, we evaluated a higher number of patients receiving evolocumab (n = 22,048) than in the previous study (n = 17,931). The observed difference in mortality trend requires cautious interpretation because we cannot fully exclude residual confounding from heterogeneity in the trial population. Another meta-analysis failed to identify an overall mortality advantage associated with the use of PCSK9i. However, alirocumab was associated with a lower risk of all-cause mortality when compared with the placebo control, but this effect was not observed with evolocumab (Guedeney et al., 2019). Furthermore, a previous study reported that evolocumab was associated with higher all-cause mortality compared to alirocumab, but the reasons for this phenomenon have not been thoroughly discussed (Wang et al., 2022).

There are concerns regarding the long-term safety outcomes of alirocumab and evolocumab. The current study did not identify overall safety issues with either drug, but two studies with the highest follow-up duration were only 2.2 years and 2.8 years (Sabatine et al., 2017; Schwartz et al., 2018). In the open-label, long-term FOURIER-OLE trial, all participants (n = 6,635) were treated with evolocumab for a median follow-up of 5.0 years. The maximum exposure to evolocumab in this trial was 8.4 years. The sustained reduction in the LDL-C levels with evolocumab was associated with reduced adverse event rates for a duration of over 8 years, and did not exceed the adverse event rates observed in the original placebo group during the parent study (O’Donoghue et al., 2022). Another clinical trial with a median follow-up period of 3.3 years enrolled patients who participated in the ODYSSEY OUTCOMES study with follow-up ranging from 3 to 5 years (n = 8,242). In this trial, the incidence rates of new-onset diabetes, worsening or complications of diabetes, and neurocognitive events were comparable between the alirocumab and placebo groups. The tolerability profile of alirocumab was comparable with the placebo, except for an a slight increase in reactions at the local injection site. During a follow-up period of 4 years, the overall occurrence of the first local injection site reaction was less than 5%, and most reactions occurred within the first 6 months (Goodman et al., 2023). The EBBINGHAUS trial specifically assessed neurocognitive safety using validation tools and demonstrated no significant differences in executive function, working memory, or psychomotor speed between evolocumab and placebo groups over a period of 19 months (Da Dalt et al., 2025). Extended follow-up in the EBBINGHAUS-OLE trial for 5.1 years further confirmed the absence of neurocognitive impairment in the ASCVD patients even when the LDL-C levels were maintained below 20 mg/dL (Zimerman et al., 2025). Several clinical trials have also demonstrated that treatment with alirocumab did not induce neurocognitive dysfunction (Kastelein et al., 2015; Leiter et al., 2017; Janik et al., 2021). Treatment with alirocumab and evolocumab did not increase the risk of hospitalization for congestive heart failure compared to placebo (1.9% vs. 1.9% with alirocumab in ODYSSEY OUTCOMES, and 2.9% vs. 3.0% with evolocumab in FOURIER) (Sabatine et al., 2017; Schwartz et al., 2018). In the extended follow-up of patients from the ODYSSEY LONG TERM study, congestive heart failure requiring hospitalization occurred in 0.6% of patients in the alirocumab group and 0.4% of patients in the placebo group, and the incidences of non-ischemic cardiac diseases were comparable between the two groups (Goodman et al., 2023). This suggested that PCSK9i were safe, effective, and well-tolerated lipid-lowering therapeutics. Long-term maintenance of very low levels of LDL-C below 20 mg per deciliter (0.5 mmol per liter) are associated with reduced risk of adverse cardiovascular events in patients with ASCVD, and these very low LDL-C levels do not show any significant safety concerns (Gaba et al., 2023). However, in primary prevention, the relative therapeutic efficacy of lowering LDL-C may decrease with age (Burger et al., 2024). It is a matter of debate whether lower LDL-C levels are associated with significant adverse clinical outcomes such as hemorrhagic stroke or new-onset diabetes. A recent review evaluated familial genetic conditions associated with lifelong, very low LDL-C levels (<30 mg/dL) and observed severe neurocognitive impairment and hepatic steatosis in abetalipoproteinemia and familial hypobetalipoproteinemia, respectively (Karagiannis et al., 2021). However, these complications were caused by mechanisms that were not related with extremely low LDL-C levels. Conversely, individuals with loss of function PCSK9 mutations or familial combined hypolipidemia maintain lifelong low LDL-C levels for decades. Individuals with loss-of-function PCSK9 mutations are healthy and do not show evidence of neurocognitive impairment, increased incidence of diabetes, cataracts, or stroke. This highlights that different genetic causes of low LDL-C levels can lead to distinct health outcomes (Karagiannis et al., 2021).

This study has a few limitations. First, this study indirectly compared the efficacy and safety of alirocumab and evolocumab because head-to-head RCT of these two drugs has not been conducted yet. Therefore, we included trials in which alirocumab or evolocumab were compared with a control group. Furthermore, clinical trials are necessary to evaluate the efficacy and safety of different types of PCSK9i in the future. Second, the experimental design varied between the studies included in this meta-analysis, especially regarding the inclusion and exclusion criteria. Third, the duration of follow-up varied between studies. For example, the follow-up duration in the EVOPACS trial was shortest among the included studies at 8 weeks, and whereas the mean follow-up duration in the FOURIER trial was 114.4 weeks. Differences in follow-up duration may introduce heterogeneity and potentially affect the effect size. Fourth, MACCE was not reported in one study. Therefore, we calculated MACCE based on the sub-outcomes reported in studies. This reconstruction approach may introduce potential biases, including variations in event definitions across different studies and the possibility of double-counting the same event. Although we made rigorous efforts to prevent duplicate counting, the results still need to be interpreted with caution. Fifth, only two types of PCSK9i were analyzed in this study. In the future, we plan to compare these with other PCSK9i or other novel lipid-lowering therapies to provide more comprehensive analysis for the clinical application of lipid-lowering regimens. Finally, most of the included trials have been previously included in earlier systematic reviews and meta-analyses of RCTs that compare alirocumab or evolocumab with the control groups. This limits the novelty of this study.

## 5 Conclusion

This updated network meta-analysis demonstrated that alirocumab and evolocumab shared a similar efficacy profile in reducing LDL-C levels. Although all-cause mortality rates were lower in ASCVD patients treated with alirocumab compared to those treated with evolocumab (RR 0.84, 95% CI 0.70–1.00), but the difference was not statistically significant. There were no significant differences in other safety endpoints between the two drugs. Compared to the placebo, both these drugs were associated with lower relative risk for MACCE, myocardial infarction, stroke, and coronary revascularization. In the future, RCTs are required to directly assess the efficacy of these two drugs on major cardiovascular events to confirm these findings and provide evidence-based guidance for clinical management.

## Data Availability

The original contributions presented in the study are included in the article/supplementary material, further inquiries can be directed to the corresponding authors.
